# Gender and age differences in KDIGO treatment targets among people on maintenance hemodialysis: Findings from a tertiary hospital in Vietnam

**DOI:** 10.1097/MD.0000000000037088

**Published:** 2024-01-26

**Authors:** Hien Van Pham, Nhat Van Tran, Truc Thanh Thai, Huong Thi Bich Tran

**Affiliations:** a Hemodialysis Department, Cho Ray Hospital, Ho Chi Minh City, Vietnam; b Ultrasonography Unit, Department of Neurology, Cho Ray Hospital, Ho Chi Minh City, Vietnam; c Department of Medical Statistics and Informatics, University of Medicine and Pharmacy at Ho Chi Minh City, Ho Chi Minh City, Vietnam; d Nephrology Division, University of Medicine and Pharmacy at Ho chi Minh City, Ho Chi Minh City, Vietnam; e Nephrology Department, Cho Ray Hospital, Ho Chi Minh City, Vietnam.

**Keywords:** age, end-stage chronic kidney disease, gender, hemodialysis, KDIGO

## Abstract

Age and gender are 2 important factors in the treatment of end-stage chronic kidney disease with hemodialysis. Understanding the influence of these 2 factors can help optimize treatment for this population. This study evaluated gender and age differences in achievement of Kidney Disease Improving Global Outcomes (KDIGO) treatment targets. A cross-sectional study was conducted on 324 chronic hemodialysis patients at a tertiary referral hospital in Ho Chi Minh City, Vietnam. KDIGO treatment targets included treatment time, prescribed Qb, treated blood volume, urea reduction ratio, spKt/V, hemoglobin, albumin, phosphorus, calcium, and parathyroid hormone. Men had significantly higher treatment time (*P* = .003), prescribed Qb (*P* = .037) and hemoglobin (*P* = .031) than women. However, women had significantly higher treated blood volume (*P* < .001), spKt/V (*P* < .001) and URR (*P* < .001). No significant difference between men and women was found in albumin, calcium, phosphorus, and parathyroid hormone. Based on KDIGO treatment targets, women had a significantly higher rate of achievement of spKt/V > 1.2 (91.4% vs 80.7%, *P* = .005) and URR ≥ 70% (77.0% vs 54.7%, *P* < .001) than men. A significantly higher rate of treated volume of ≥ 1 L/kg/BW, and phosphorus 2.5 to 4.6 mg/dL was found in women (90.0% and 40.2%) compared to men (68.7% and 27.3%). In contrast, men had a significantly higher rate of prescribed Qb ≥ 300 mL/min (26.7% vs 12.6%, *P* = .001), albumin ≥ 40 g/L (36.7% vs 26.4%, *P* = .047), and Hb > 12 g/dL (22.0% vs 11.5%, *P* = .011) than women. There was no significant difference between men and women in the rate of calcium 8.4 to 10.4 mg/dL, and parathyroid hormone 150 to 600 pg/mL. These differences were not the same across 4 age categories (<60, 60–69, 70–79, and ≥ 80). Most of the differences above were among patients aged < 60 and 60 to 69 years. Although men had higher satisfactory treatment parameters than women, based on KDIGO treatment targets, women received hemodialysis more effectively than men. Treatment targets for patients on hemodialysis should consider gender and age differences.

## 1. Introduction

According to the United States Renal Data System for 2001 to 2021, the incidence of end-stage renal disease in men was much higher than that in women.^[1]^ However, women often initiate dialysis later than men.^[2]^ Under regular maintenance hemodialysis, women have a higher risk of hospitalization and lower survival rate compared to men.^[3,4]^ It is more difficult to create and easy to fail an arteriovenous fistula for women compared to men, although routine vascular mapping is used and monitored.^[5]^ Although Kidney Disease Improving Global Outcomes (KDIGO) applies the same target values to both men and women for treating anemia and chronic kidney disease-mineral and bone disorder, many studies have shown gender differences regarding the treatment of this disease.^[6,7]^ Therefore, understanding such differences can be beneficial in optimizing treatment for this population.

While a large body of literature has revealed the importance of aging in treatment and prognosis of patients on hemodialysis, the gender differences also vary significantly across different age groups. In a study among patients undergoing maintenance hemodialysis, Carrero et al (2011)^[8]^ found that women aged < 45 years had a significantly higher mortality rate than men. However, according to United States Renal Data System 2022, the older population had a higher mortality rate but no difference between men and women was found.^[1]^ As age increases, the number of patients starting hemodialysis at an eGFR < 10 mL/min/1.73m^2^ decreases in both sexes.^[1]^ In clinical practice, treatment and hemodialysis prescription for the elderly could be different from younger patients. For example, the vascular access types used in hemodialysis in the elderly were influenced by age and gender. Prescribed treatment time and prescribed blood flow were significantly different between the elderly and younger age group.^[9]^ Therefore, the differences in the adequacy and effectiveness of treatment and hemodialysis prescription between men and women across age groups warrant further investigation in specific populations.

In Vietnam, there are approximately 30,000 hemodialysis patients,^[10]^ with 53.8% women and 46.2% men. To date, there are no studies on gender and age differences in the treatment of end-stage chronic kidney disease with routine hemodialysis. All studies conducted in Vietnam have mainly focused on evaluating the effectiveness of hemodialysis adult populations in overall. There is a need to evaluate treatment parameters as recommended by KDIGO^[11]^ based on age and gender, so that the most appropriate treatment method can be determined. Therefore, this study evaluated the gender and age differences in achievement of KDIGO targets in terms of hemodialysis practices including hemodialysis parameters, dialysis adequacy, treatment of renal anemia and CKD–MBD.

## 2. Materials and methods

A cross-sectional study was conducted on chronic hemodialysis patients at Cho Ray Hospital, Ho Chi Minh City, Vietnam. This hospital is a tertiary referral hospital providing hemodialysis for a total of 350 hemodialysis patients coming from all provinces and cities in the country. At the study hospital, over 90% of the vascular access used for hemodialysis are mainly arteriovenous fistula, the rest are cuffed tunnel catheters, and arteriovenous graft (AVG) accounts for a very low percentage. All patients are on hemodialysis 3 times a week for a dialysis time of at least 4 hours each time. During the study period, a total of 324 hemodialysis patients agreed to participate in the study on voluntary basis. The study was approved by the Ethics Committee for Biomedical Research, University of Medicine and Pharmacy at Ho Chi Minh City (320/HĐĐĐ-ĐHYD, 12th May 2020), and Cho Ray Hospital.

Age, gender, body mass index (BMI), and the number of dialysis vintage years were collected from interviewing patients using a questionnaire. The hemodynamic parameters were collected during hemodialysis (at least 30 minutes), at the second hemodialysis shift. All patient biochemical data were also collected. All laboratory analyses met Conformité Européenne - In vitro Diagnostics quality standards. Except for spKt/v and URR, which were calculated using formula, all remaining parameters were measured directly in blood including hemoglobin (Hb), urea, albumin, calcium, phosphorus, and parathyroid hormone.

The single-pool urea Kt/V (spKt/V) was calculated by formula: spKt/V = -ln(*R*-0,008xt) + (4–3,5xR) × 0,55 UF/V. In which, Kt/V = 2,2-(3,3x(R-(0,03-UF/W))), R = post dialysis BUN (blood urea nitrogen) from venous line; predialysis BUN from arterial line; UF: ultrafiltration (kg); W: body weight post dialysis (kg); K: Urea clearance of dialyzer (L/h); t: dialysis time (h); V: Urea distribution volume (L).^[12]^ Urea reduction ratio (URR) = 100% × (predialysis BUN-postdialysis BUN)/predialysis BUN.

Data were analyzed using Stata 16.0 (Stata Corporation, College Station, TX, USA). Quantitative data were presented in the form of mean and standard deviation, or median and interquartile range, depending on data distribution. Counts and percentages were used to present qualitative data. The difference between men and women was tested using Student *t* test, Mann-Whitney test, Chi-squared test, Fisher exact test when appropriate. This difference was also examined in 4 age categories including < 60, 60 to 69, 70 to 79, and ≥ 80. A *P* value of <.05 was considered statistically significant.

## 3. Results

Table 1 presents a comparison of demographic data, hemodialysis prescription and laboratory parameters related to hemodialysis adequacy, renal anemia and CKD–MBD between women and men. There were significant differences in body weight, treatment time, prescribed Qb, treated blood volume, spKt/V, URR, Hb and interdialytic body weight gain between men and women. Men had significantly higher body weight (*P* < .001), treatment time (*P* = .003), prescribed Qb (*P* = .037), Hb (*P* = .031) and interdialytic body weight gain (*P* < .001) than women. However, women had significantly higher treated blood volume (*P* < .001), spKt/V (*P* < .001) and URR (*P* < .001). No significant difference between men and women was found in age, dialysis vintage, BMI, albumin, calcium, phosphorus, PTH, mean arterial pressure predialysis and diabetes mellitus.

**Table 1:** Gender differences in general characteristics, prescription of hemodialysis, and laboratory parameters among hemodialysis patients (n = 324).

	Women n* =* 174 (53.7%) *M (SD*)	Men n* = *150 (46.3%) M (SD)	*P* value
General characteristics			
Age (yr)	51.2 (15.5)	48.1 (14.6)	.066
Dialysis vintage (mo) (median, IQR)	54.2 (20.1–105.2)	54.7 (24.0–120.2)	.420
Body weight (kg)	50.1 (9.3)	59.9 (12.3)	<.001
Body mass index (kg/m^2^)	21.1 (4.5)	21.9 (4.3)	.148
Mean arterial pressure predialysis (mm Hg)	99.3 (10.3)	98.0 (9.5)	.231
CVC (%) n (%)	87 (50.0)	74 (49.3)	.905
Diabetes mellitus n (%)	20 (15.9)	20 (14.9)	.617
Hemodialysis session			
Treatment time (h)	3.8 (0.3)	3.9 (0.2)	.003
Prescribed Qb (mL/min)	165.1 (23.5)	271.1 (27.6)	.037
Treated blood volume (L/kg)	1.3 (0.3)	1.1 (0.2)	<.001
Interdialytic body weight gain (kg)	2.3 (0.8)	2.6 (0.9)	<.001
Urea reduction ratio (%)	74.9 (9.2)	69.8 (8.3)	<.001
spKt/V	1.7 (0.4)	1.5 (0.4)	<.001
Laboratory parameters			
Hemoglobin (g/dL)	9.9 (2.1)	10.4 (2.0)	.031
Albumin (g/L)	37.5 (3.8)	38.1 (3.5)	.113
Calcium (mg/dL)	9.0 (0.8)	9.0 (1.1)	.810
Phosphorus (mg/dL)	5.3 (3.1)	5.6 (1.9)	.315
Parathyroid hormone (pg/mL) (median, IQR)	271.5 (117.0–562.0)	304.5 (135.5–701.0)	.341

**Figure 1.**
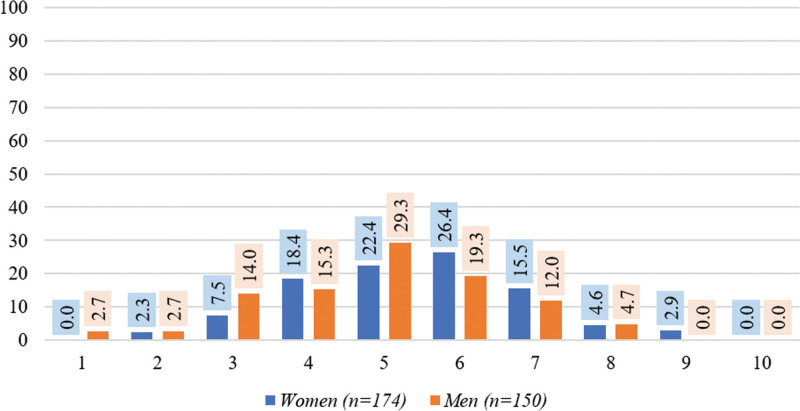
The number of KDIGO treatment targets achieved in men and women on hemodialysis (*P* = .014).

Based on KDIGO treatment targets of spKt/V > 1.2 or URR ≥ 70%, women had a significantly higher rate of achievement of treatment target than men in both spKt/V (91.4% vs 80.7%, *P* = .005) and URR (77.0% vs 54.7%, *P* < .001) (Table 2). Also, a higher rate of treated volume of ≥ 1 L/kg, and phosphorus 2.5 to 4.6 mg/dL was found in women (90.0% and 40.2%) compared with men (68.7% and 27.3%). In contrast, men had a significantly higher rate of prescribed Qb ≥ 300 mL/min (26.7% vs 12.6%, *P* = .001), albumin ≥ 40 g/L (36.7% vs 26.4%, *P* = .047), and Hb > 12 g/dL (22.0% vs 11.5%, *P* = .011) than women. There was no significant difference between men and women in the rate of Hb 10 to 12 g/dL, calcium 8.4 to 10.4 mg/dL, and PTH 150 to 600 pg/mL. The number of KDIGO treatment targets was significantly different between men and women (*P* = .014) (Fig. 1).

**Table 2:** Gender differences in achievement of KDIGO treatment targets among hemodialysis patients (n = 324).

	Treatment targets	Women n* =* 174 (53.7%) n (%)	Men n* = *150 (46.3%) n (%)	*P* value
1	Treatment time ≥ 12 h/wk	91 (52.3)	81 (54.0)	.760
2	Prescribed Qb ≥ 300 mL/min	22 (12.6)	40 (26.7)	.001
3	Treated volume ≥ 1 L/kg/BW	156 (90.0)	103 (68.7)	<.001
4	Urea reduction ratio ≥ 70%	134 (77.0)	82 (54.7)	<.001
5	spKt/V > 1.2	159 (91.4)	121 (80.7)	.005
6	Hemoglobin 10–12 g/dL	68 (39.1)	57 (38.0)	.842
	Hemoglobin* *> 12 g/dL	20 (11.5)	33 (22.0)	.011
7	Albumin ≥ 40 g/L	46 (26.4)	55 (36.7)	.047
8	Phosphorus 2.5–4.6 mg/dL	70 (40.2)	41 (27.3)	.015
9	Calcium 8.4–10.4 mg/dL	118 (67.8)	100 (66.7)	.826
10	Parathyroid hormone 150–600 pg/mL	80 (46.0)	63 (42.0)	.472

Table 3 presents a comparison of demographic data, hemodialysis prescription and laboratory parameters related to hemodialysis adequacy, renal anemia and hronic kidney disease-mineral and bone disorder between men and women across 4 age categories including < 60, 60 to 69, 70 to 79, and ≥ 80. The difference between men and women in body weight and treated blood volume was similar across age categories where men had significantly higher body weight and lower treated blood volume than women. However, other differences, as shown in Table 1, were not consistent across the 4 age categories. The difference between men and women was only observed in treatment time among those aged 60 to 69 years (*P* = .005), in prescribed Qb among those aged 60 to 79 years, in spKt/V among those aged < 60 years and in interdialytic body weight gain among those aged < 69 years.

**Table 3:** Gender differences in general characteristics, prescription of hemodialysis, and laboratory parameters among hemodialysis patients across different age categories (n = 324).

Patient characteristics	< 60 (n = 227)			60–69 (n = 65)			70–79 (n = 25)			≥ 80 (n = 8)		
	Women	Men	*P* value	Women	Men	*P* value	Women	Men	*P* value	Women	Men	*P* value
General characteristics												
Age, mean ± SD (yr)	42.0 ± 10.1	41.9 ± 9.9	.911	63.9 ± 2.6	63.9 ± 2.6	.966	73.8 ± 3.1	73.7 ± 2.1	.897	83.0 ± 2.6	85.0 ± 2.0	.267
Dialysis vintage, median (IQR) (mo)	60.1 (26.1–120.1)	69.0 (28.4–128.6)	.446	60.1 (23.0–93.4)	47.6 (34.1–78.1)	.525	45.1 (18.1–67.1)	15.5 (6.6–23.0)	.203	11.1 (7.1–19.9)	41.6 (26.6–101.7)	.043
Body weight, mean ± SD (kg)	48.7 ± `8.8	58.9 ± 12.4	<.001	53.5 ± 8.3	65.7 ± 11.6	<.001	53.4 ± 11.5	55.4 ± 6.9	.686	41.9 ± 1.8	58.4 ± 8.0	.023
Body mass index, mean ± SD (kg/m^2^)	20.3 ± 3.7	21.6 ± 4.5	.020	23.1 ± 5.9	23.6 ± 3.1	.685	22.7 ± 4.4	20.9 ± 2.4	.340	19.7 ± 1.4	20.4 ± 3.0	.657
Mean arterial pressure predialysis, mean ± SD (mm Hg)	100.2 ± 11.2	98.5 ± 10.0	.213	97.2 ± 8.0	96.4 ± 8.7	.716	97.2 ± 8.2	96.7 ± 2.1	.801	104.2 ± 12.0	96.7 ± 6.7	.316
CVC (%) n (%)	55 (48.7)	53 (46.5)	.742	17 (44.7)	15 (57.7)	.446	13 (68.4)	2 (33.3)	.175	2 (50.0)	4 (100.0)	.429
Diabetes mellitus n (%)	9 (8.0)	10 (8.8)	1.000	5 (13.2)	6 (23.1)	.331	5 (26.3)	3 (50.0)	.344	1 (25.0)	1 (25.0)	1.000
Hemodialysis session												
Treatment time, mean ± SD (h)	3.8 ± 0.03	3.9 ± 0.02	.090	3.8 ± 0.3	4.0 ± 0.1	.005	3.7 ± 0.3	3.8 ± 0.3	.779	3.6 ± 0.3	3.8 ± 0.3	.537
Prescribed Qb, mean ± SD (mL/min)	269.2 ± 24.2	273.1 ± 27.7	.266	259.7 ± 20.1	272.3 ± 24.4	.028	257.4 ± 15.2	238.3 ± 24.8	.032	237.5 ± 35.0	257.5 ± 15.0	.334
Treated blood volume, mean ± SD (L/kg)	1.4 ± 0.3	1.2 ± 0.2	<.001	1.2 ± 0.2	1.0 ± 0.2	<.001	1.2 ± 0.2	1.04 ± 0.2	.141	1.4 ± 0.2	1.1 ± 0.2	.042
Interdialytic body weight gain, mean ± SD	2.3 ± 0.8	2.7 ± 0.9	.002	2.2 ± 0.8	2.7 ± 0.7	.015	2.0 ± 0.7	2.0 ± 1.2	.968	1.4 ± 0.5	2.2 ± 0.9	.145
Urea reduction ratio (%)	75.7 ± 9.5	70.3 ± 8.4	<.001	74.5 ± 7.5	68.6 ± 8.4	.004	72.3 ± 10.5	65.4 ± 7.8	.152	65.2 ± 3.2	70.6 ± 4.7	.105
spKt/V, mean ± SD	1.8 ± 0.4	1.5 ± 0.4	<.001	1.7 ± 0.3	1.4 ± 0.3	.006	1.6 ± 0.5	1.3 ± 0.2	.152	1.2 ± 0.1	1.4 ± 0.1	.081
Laboratory parameters												
Hemoglobin, mean ± SD (g/dL)	9.9 ± 2.2	10.4 ± 2.1	.087	9.9 ± 1.9	10.9 ± 2.1	.054	10.2 ± 1.4	9.7 ± 1.2	.444	10.5 ± 1.1	9.8 ± 0.8	.359
Albumin, mean ± SD (g/L)	37.7 ± 3.9	38.5 ± 3.6	.109	37.4 ± 3.3	37.2 ± 3.3	.784	37.1 ± 4.0	36.5 ± 2.6	.730	35.5 ± 4.0	37.8 ± 2.6	.387
Calcium, mean ± SD (mg/dL)	9.1 ± 0.8	8.9 ± 1.2	.369	9.0 ± 0.8	9.3 ± 0.9	.217	8.9 ± 0.9	8.8 ± 0.8	.783	8.5 ± 1.0	8.9 ± 0.8	.526
Phosphorus, mean ± SD (mg/dL)	5.4 ± 3.6	5.8 ± 1.9	.297	5.0 ± 1.9	4.9 ± 1.6	.679	5.3 ± 1.7	5.0 ± 1.1	.701	4.6 ± 1.1	4.3 ± 2.6	.880
PTH, median (IQR) (pg/mL)^a^	333.0 (129.7–612.2)	372.6 (155.6–831.0)	.190	225.5 (84.7–558.0)	184.3 (93.9–349.0)	.667	189.9 (146.4–494.0)	140.2 (80.5–161.0)	.227	261.4 (143.6–311.0)	177.4 (77.1–278.0)	.387

**Figure 2.**
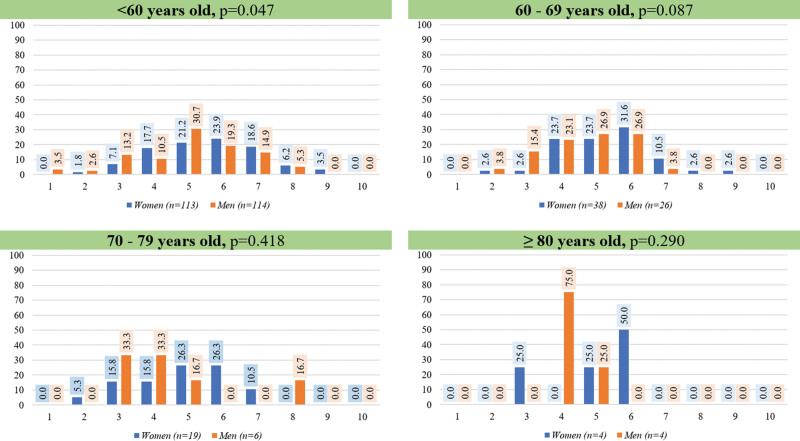
The number of KDIGO treatment targets achieved in men and women on hemodialysis across 4 age categories.

The rates of achievement of KDIGO treatment targets between men and women across 4 age categories are shown in Table 4. The differences between men and women in spKt/V > 1.2, URR ≥ 70%, prescribed Qb ≥ 300 mL/min, treated blood volume ≥ 1 L/kg and phosphorus < 5.5 mg/dL as presented in Table 1 were only observed in those aged < 60 years. For those aged 60 to 69 years, the differences between men and women remained significant in URR ≥ 70% and treated blood volume ≥ 1 L/kg where women had a higher rate than men. There was no significant difference in any KDIGO treatment targets between men and women aged 70 years or above. The number of KDIGO treatment targets was marginally statistically different between men and women aged ≤ 69 years, but not for those aged ≥ 70 years (Fig. 2).

**Table 4:** Gender differences in achievement of KDIGO treatment targets among hemodialysis patients across different age categories (n = 324).

KDIGO treatment targets		< 60 (n = 227)			60–69 (n = 64)			70–79 (n = 25)			≥ 80 (n = 8)		
		Women n (%)	Men n (%)	*P* value	Women n (%)	Men n (%)	*P* value	Women n (%)	Men n (%)	*P* value	Women n (%)	Men n (%)	*P* value
1	Treatment time ≥ 12 h/wk	62 (54.9)	66 (57.9)	.646	21 (55.3)	11 (42.3)	.446	6 (31.6)	4 (66.7)	.175	2 (50.0)	0 (0.0)	.429
2	Prescribed Qb ≥ 300 mL/min	18 (15.9)	35 (30.7)	.009	3 (7.9)	5 (19.2)	.253	1 (5.3)	0 (0.0)	1.000	-	-	-
3	Treated blood volume ≥ 1L/kgBW	106 (93.8)	83 (72.8)	<.001	32 (84.2)	14 (53.9)	.011	14 (73.7)	3 (50.0)	.344	4 (100.0)	3 (75.0)	1.000
4	Urea reduction ratio ≥ 70%	94 (83.2)	65 (57.0)	<.001	28 (73.7)	12 (46.2)	.036	12 (63.2)	3 (50.0)	.653	0 (0.0)	2 (50.0)	.429
5	spKt/V > 1.2	107 (94.7)	94 (82.5)	.003	35 (92.1)	20 (76.9)	.142	14 (73.7)	3 (50.0)	.344	3 (75.0)	4 (100.0)	1.000
6	Hemoglobin 10–12 g/dL	39 (34.5)	39 (34.2)	.962	17 (44.7)	13 (50.0)	.800	9 (47.4)	3 (50.0)	1.000	3 (75.0)	2 (50.0)	1.000
	Hemoglobin* *> 12 g/dL	15 (13.3)	26 (22.8)	.062	4 (10.5)	7 (26.9)	.104	1 (5.3)	0 (0.0)	1.000	-	-	-
7	Albumin ≥ 40g/L	32 (28.3)	47 (41.2)	.041	8 (21.1)	6 (23.1)	1.000	6 (31.6)	1 (16.7)	.637	0 (0.0)	1 (25.0)	1.000
8	Phosphorus 2.5–4.6 mg/dL	44 (38.9)	26 (22.8)	.009	17 (44.7)	11 (42.3)	1.000	6 (31.6)	3 (50.0)	.630	3 (75.0)	1 (25.0)	.486
9	Calcium 8.4–10.4 mg/dL	78 (69.0)	76 (66.7)	.703	27 (71.1)	17 (65.4)	.784	11 (57.9)	5 (83.3)	.364	2 (50.0)	2 (50.0)	1.000
10	Parathyroid hormone 150–600 pg/mL	49 (43.4)	46 (40.4)	.646	15 (39.5)	13 (50.0)	.450	13 (68.4)	2 (33.3)	.175	3 (75.0)	2 (50.0)	1.000

## 4. Discussion

This study is among the first in Vietnam to investigate gender differences across different age categories in hemodialysis, laboratory parameters and KDIGO treatment targets among a large sample of patients on maintenance hemodialysis. We found significant differences between men and women in general characteristics (i.e., body weight), all characteristics of hemodialysis session and laboratory parameters (i.e., hemoglobin). However, these differences were not the same across age categories.

In our study, there were no significant differences between men and women in general characteristics except body weight where men had a higher body weight than women. However, BMI was not different between men and women. Our study was similar to Weigert et al ‘s study (2020) where body weight was different between men and women, but BMI was not.^[13]^ Moreover, in our study, all metrics in the hemodialysis session were significantly different between men and women. We found that the mean treatment time and blood flow rate in men were higher than those in women. Long time hemodialysis and high flow blood rate will cause uncomfortable and high heart rate. Women often have poor tolerance to this condition. Hecking et al (2014) reported similar results,^[4]^ however, in the study by Weigert et al (2020) while the blood flow rate of men was higher than women, hemodialysis time was not different between men and women.^[13]^ In terms of interdialytic weight gain, in our study, men gained more than women which is similar to a study by Ifudu et al (2002).^[14]^ Women body weight was lower than men, and thus, treated blood volume women (based on weight) was higher than men. As a result, the adequacy hemodialysis (spKt/V and URR) in women was higher than that in men.

Conventional hemodialysis remains the most common treatment for kidney replacement in Vietnam and is usually performed for 4 hours, 3 days per week (minimum 12 hours per week) regardless of gender and age. In our study, all treatment targets based on KDIGO were significantly different between men and women. Although there were more women treated with a cleaned blood volume ≥ 1 L/kg than men, the number of women achieving a blood flow rate of ≥ 300 mL/min was lower than that of men.^[15]^ Therefore, the rate of achieving URR and spKt/V was higher in women than in men. Our findings were supported by previous studies where the level of adequacy hemodialysis in women was higher than men.^[13]^ This is important because adequacy hemodialysis is a factor that contributes to the survival benefit of women compared with men.^[16]^

Renal anemia is a key factor in the management of hemodialysis patients. In the present study, although hemoglobin level of 10 to 12 g/dL was not significantly different between men and women, the number of patients with hemoglobin levels > 12 g/dL in men was higher than that in women. In the general population, women have lower hemoglobin levels than men, and the definition of anemia is sex-specific.^[17]^ Hemoglobin levels remain lower in women across all stages of CKD, however, the KDIGO dialysis treatment guidelines do not consider gender-specific targets.^[18]^ Treatment of anemia in women requires higher doses of erythropoiesis stimulating agents than in men, and women are less responsive to erythropoiesis stimulating agents than men. However, in the KDIGO guidelines, there was no difference in weekly erythropoiesis stimulating agents doses between women and men at any age.^[19,20]^ Moreover, in adults with normal kidney function, serum phosphate levels in women are higher than in men, and gradually increase with age.^[21]^ In our study, phosphorus levels increased both in men and women, but the number of women achieving phosphate 2.5 to 4.6 mg/dL (KDIGO) was higher than men. This may be because the cleaned blood volume (treated volume ≥ 1 L/kg) of women was higher than that of men.

Gender differences across age groups are important so that treatment for each population can be optimized. In our study, age and dialysis vintage were not different between men and women. However, studies from Dialysis Outcomes and Practice Patterns Study have shown that patient age and dialysis vintage differed slightly between sexes.^[4]^ In our study, body weight was different between men and women only among those aged ≤ 69 and ≥ 80 years. The difference in BMI between sexes was only observed in < 60 years old group. It is possible that for those aged over 60 years, osteodystrophy plus bone pathology caused by ESKD make the difference no longer different. In the Dialysis Outcomes and Practice Patterns Study analysis, women also had a higher BMI than men.^[4]^ In our study, treatment time between men and women in the 60 to 69-year-old group was also different. There is little evidence that hemodialysis should be administered at least every 4 hours for all patients, regardless of body weight, solute removal, or residual renal function.^[22]^ In the groups < 60 and ≥ 80 years old, blood flow rate during hemodialysis was not significantly different, but in the groups 60 to 69 and 70 to 79 years old, men had a higher blood flow than women. It is possible that in the group < 60 years old, most men and women can tolerate well with a high blood flow rate, and in the group ≥ 80 years old, both had a low blood flow rate during hemodialysis. In addition, in elderly patients with CKD, comorbidities become more and more prevalent^[23]^ and women tolerance to these conditions is lower than that of men.

In terms of interdialytic weight gain, men gain more weight than women, but this difference is only statistically significant among those aged ≤ 69 years. The reason may be that for those aged over 70 years, malnutrition is similar in both sexes. For spKt/V and URR, the difference between men and women is only significant in those < 60 years old where women received more adequacy hemodialysis than men. A previous study by Weigert et al (2020) also reported that there was no gender difference in hemodialysis adequacy across age groups, except those aged 80 or more years.^[13]^ In contrast, a study by Rezaiee et al (2016) indicated that there was a significant correlation between hemodialysis adequacy and age, gender.^[24]^

There are several implications of our findings. First, although men have higher satisfactory treatment parameters than women, based on KDIGO treatment targets, women received hemodialysis more effectively than men. The differences found in our study indicate that the treatment target should be gender-specific because all available clinical guidelines apply the same target values for both men and women. Second, while the gender differences not remaining significant across age groups in our study require further investigation to confirm, this suggests that age should be considered in clinical treatment for both sexes. Third, the similarity in the number of KDIGO treatment targets in men and women reveals that when considering gender and age, more weight should be used in the calculation to prioritize important targets than the others.

Our study had some limitations. First, this study was conducted at only 1 tertiary hospital and thus the results might not be generalized to this population in other settings where the treatment approach and facilities may be different. Second, the study only evaluated gender and age differences in hemodialysis patients using arteriovenous fistula and did not represent the entire hemodialysis patient population where arteriovenous graft or catheter hemodialysis were used. Third, the results were only evaluated through 1 hemodialysis session, while ideally, they needed to be evaluated multiple times to make the results more objective and accurate. Fourth, evaluating hemodialysis adequacy through URR or spKt/V is based on blood urea samples at different times of membrane reuse, which may have bias. Moreover, although the effectiveness of hemodialysis sessions measured in our study is important, this should be linked to long-term treatment outcomes such as mortality and complications. Therefore, more studies are needed to evaluate the effect of gender and age differences in hemodialysis sessions on long-term treatment outcomes.

## 5. Conclusions

Although men had higher satisfactory treatment parameters than women, based on KDIGO treatment targets, women received hemodialysis more effectively than men. However, these differences were not the same across age categories. Therefore, treatment targets for patients on hemodialysis should consider gender and age differences.

## Acknowledgments

The authors thank all the patients who participated in this study. We also thank the director board of Cho Ray hospital for their support during the study.

## Author contributions

**Conceptualization:** Hien Van Pham, Truc Thanh Thai, Huong Thi Bich Tran.

**Data curation:** Hien Van Pham, Nhat Van Tran, Huong Thi Bich Tran.

**Formal analysis:** Hien Van Pham, Truc Thanh Thai.

**Supervision:** Huong Thi Bich Tran.

**Writing – original draft:** Hien Van Pham, Nhat Van Tran, Truc Thanh Thai, Huong Thi Bich Tran.

**Writing – review & editing:** Hien Van Pham, Nhat Van Tran, Truc Thanh Thai, Huong Thi Bich Tran.
